# Microbial and Functional Profile of the Ceca from Laying Hens Affected by Feeding Prebiotics, Probiotics, and Synbiotics

**DOI:** 10.3390/microorganisms7050123

**Published:** 2019-05-06

**Authors:** Carolina Pineda-Quiroga, Daniel Borda-Molina, Diego Chaves-Moreno, Roberto Ruiz, Raquel Atxaerandio, Amélia Camarinha-Silva, Aser García-Rodríguez

**Affiliations:** 1 Neiker-Tecnalia, Department of Animal Production, Granja Modelo de Arkaute, Vitoria-Gasteiz 01080, Spain; cpineda@neiker.eus (C.P.-Q.); rruiz@neiker.eus (R.R.); ratxaerandio@neiker.eus (R.A.); 2 University of Hohenheim, Institute of Animal Science, Department of Livestock Microbial Ecology, 70599 Stuttgart, Germany; daniele.borda@uni-hohenheim.de (D.B.-M.); amelia.silva@uni-hohenheim.de (A.C.-S.); 3 Helmholtz Centre for Infection Research, Microbial Interactions and Processes Research Group, 38124 Braunschweig, Germany; Diego.Chaves-Moreno@helmholtz-hzi.de

**Keywords:** poultry, lactose, 16S rRNA amplicon sequencing, metagenome sequencing

## Abstract

Diet has an essential influence in the establishment of the cecum microbial communities in poultry, so its supplementation with safe additives, such as probiotics, prebiotics, and synbiotics might improve animal health and performance. This study showed the ceca microbiome modulations of laying hens, after feeding with dry whey powder as prebiotics, *Pediococcus acidilactici* as probiotics, and the combination of both as synbiotics. A clear grouping of the samples induced per diet was observed (*p* < 0.05). Operational taxonomic units (OTUs) identified as *Olsenella* spp., and *Lactobacillus* *crispatus* increased their abundance in prebiotic and synbiotic treatments. A core of the main functions was shared between all metagenomes (45.5%), although the genes encoding for the metabolism of butanoate, propanoate, inositol phosphate, and galactose were more abundant in the prebiotic diet. The results indicated that dietary induced-changes in microbial composition did not imply a disturbance in the principal biological roles, while the specific functions were affected.

## 1. Introduction

It is widely recognized that diet is one of the contributing factors shaping the composition and functions encoded by the microbiota in the gastrointestinal tract (GIT) of poultry [1,2,3]. Dietary supplements, such as prebiotics, probiotics, and synbiotics, have frequently been used in the poultry industry as a feasible alternative to increase animal health and performance [4]. Prebiotics are substances acting as growth substrates for microorganisms harboring the digestive tract, thus, promoting the growth and establishment of beneficial bacteria, enhancing their activity and positively affecting the host [5]. Probiotics refer to the directly fed microorganisms, which affect the host, either directly or through their products, or even by the influence on other microbial activities [6]. The mixture of both components was also used, turning them into synbiotics, which conferred benefits beyond those achieved by themselves [7].

The ceca were the primary sites of fermentation of the avian GIT, and it harbored the most complex and yet not entirely characterized microbial community [8,9]. Ceca resident microorganisms were responsible for a wide range of catabolic pathways, resulting in the synthesis of a variety of products that were accessible to the host [10]. For instance, short chain fatty acids, such as fermentation products [11], synthesis of vitamins B and K [12], and nitrogen recycling by the breakdown of uric acid [13], were some of the main products attributed to the microbial activity. A better understanding of the phylogenetic structure and functional capacity of the ceca microbial consortia, in response to dietary interventions, is essential to elucidate their roles in the host physiology and productivity.

Over the last two decades, culture-independent methods have been developed to overcome the cultivation biases, offering detailed information about the microbial composition, diversity structure, and functionality [14]. Sequencing of the 16S ribosomal RNA gene has served as a useful tool for taxonomy classification [15] and has been applied for investigating the poultry intestinal microbiota, in depth [1,2,3]. On the other hand, the wide variation in the genome content, between even closely related strains of microbes, makes it challenging to assign the functions to microbial populations based on 16S sequence inventories alone [16]. For this reason, environmental and community genomics, generally referred to as “metagenomics,” should be applied to enhance the knowledge of microbial functions. As a result, the identification of the gene content of the microorganisms and their biological functions within an ecosystem, could be determined, and a catalog of genes was established [17,18].

Therefore, this study aimed to give an insight into the ceca microbiota changes, in response to the dietary inclusion of prebiotic dry whey powder (DWP), probiotic *Pediococcus acidilactici* (PA), and a mixture of both, through the analysis of the microbial community structure and function. The obtained results indicate that each dietary supplementation strongly influenced the ceca microbial consortia, even though these changes did not imply a disturbance in the main biological roles encoded by the bacteria community.

## 2. Materials and Methods

### 2.1. Ethical Statement

This experiment was performed in accordance with the European Union (2010/63/EU) and Spanish regulations (RD 53/2013), for the care and use of animals for experimental and other scientific purposes.

### 2.2. Animals and Experimental Diets

The study was conducted at the experimental farm of Neiker-Tecnalia in Arkaute (Vitoria-Gasteiz, Spain). Laying hens were managed and fed with the diets described by Pineda-Quiroga et al. [19]. Briefly, a flock of 300, 57-week-old hens (ISA Brown strain, Avigán Terralta S.A, Tarragona, Spain), allocated in floor pens, were used in an experiment, for 70 days. Birds were randomly assigned to separate pens, with 15 birds in each pen, and five pens per treatment. The diets involved a control diet (no supplementation of dry whey powder-(DWP) or *Pediococcus acidilactici* (PA)), prebiotic (60 g/kg of addition of DWP), probiotic (2 g/kg of addition of PA), and synbiotic (a mixture of 60 g/kg of addition of DWP and 2 g/kg of addition of PA). The dry whey powder used was a commercial sweet powder (Sueromancha S.L, Toledo, Spain; 703 g of lactose/kg of product) and the probiotic was a commercial probiotic “Bactocell” (Lallemand, France), containing a live culture of *P. acidilactici* (strain MA 18/5, 1010 CFU/g). Feed and water were provided ad libitum, throughout the experiment.

### 2.3. Cecal Sample Collection and DNA Extraction

On the last day of the experiment, 12 hens (three per treatment) were randomly selected from different pens and euthanized by CO_2_ inhalation to extract the ceca digesta content. Samples were immediately stored at −80 °C, until further analysis. The total nucleic acid was obtained using the PowerSoil DNA extraction Kit (MOBIO Laboratories Inc., Carlsbad, CA, USA), according to the manufacturer recommendations.

### 2.4. 16S rRNA Gene Amplification, Sequencing, and Analysis

Cecal DNA from the sampled hens was used for the Illumina amplicon library preparation. PCR amplification of the V1-2 hypervariable region of the 16S rRNA gene was performed according to Kaewtappe et al. [20]. Libraries were sequenced using 250 base pairs paired-end sequencing chemistry, on an Illumina MiSeq platform.

Bioinformatic processing of the Illumina reads, followed the Mothur-Miseq SOP [21]. Sequences were aligned using the SILVA-based bacterial reference alignment, and chimera sequences were checked and removed using UCHIME [21]. Reads were then clustered into operational taxonomic units (OTUs), using a ≥97% sequence similarity threshold. Finally, a total of 934 OTUs were taxonomically assigned using the naïve Bayesian RDP classifier. Sequences are available at the European Nucleotide Archive, under the accession number PRJEB21237 in http://www.ebi.ac.uk/ena/data/view/PRJEB21237.

Relative abundances per out, on each sample, were analyzed using multivariate statistical routines in PRIMER, considering the Bray–Curtis similarity coefficient (version 7.0.9, PRIMER-E; Plymouth Marine Laboratory, Plymouth UK; [22]). The microbial community structure was explored by a non-metric multidimensional scaling plot (nMDS), and the statistical comparison between diets was determined through a permutational analysis of variance (PERMANOVA, 999 permutations). Pielou’s evenness index and Shannon-weaver index of diversity (H’) were determined and analyzed using a Kruskal–Wallis test (R environment, V 3.3.3).

### 2.5. Metagenome Sequencing and Analysis

Cecal DNA from one laying hen per diet was sequenced through the Illumina HiSeq2500 platform. The sequencing generated an average number of 7′528.949 sequences, with a length of 100 base pairs, which were cleaned and assembled using the CLC Main Workbench software version 9.0.1 (CLCbio^®^, Germantown, MD, USA). Subsequently, gene annotations were done through the metagenomics RAST server version 4 (MG-RAST; [23]). The KEGG database for proteins taxonomic assignment and the KEGG orthologs (KO) for the annotation analysis were considered, working with the MG-RAST parameters, by default. The metagenome sequences were publicly available under the MG-RAST project mgp21245 (Metagenome IDs: control [mgm4730023.3], probiotic [mgm4730065.3], prebiotic [mgm4730022.3], and synbiotic [mgm4730024.3]).

For the data analysis, the log_2_fold change (LFC) in the presence of genes was calculated, based on normalized reads with the DESeq2, R package. Genes meeting the cut-off criteria of *p*-value ≤ 0.05 (Wald test), LFC ≥ 1, or LFC ≤ −1, were considered to be differentially present. Venn diagram was depicted with the genes shared between each diet and their interactions, using the online tool Venny 2.1.0 (Available online: http://bioinfogp.cnb.csic.es/tools/venny/index.html). 

## 3. Results

### 3.1. Microbial Community Analysis Based on 16S rRNA Gene Amplicon Sequencing

**Figure 1:**
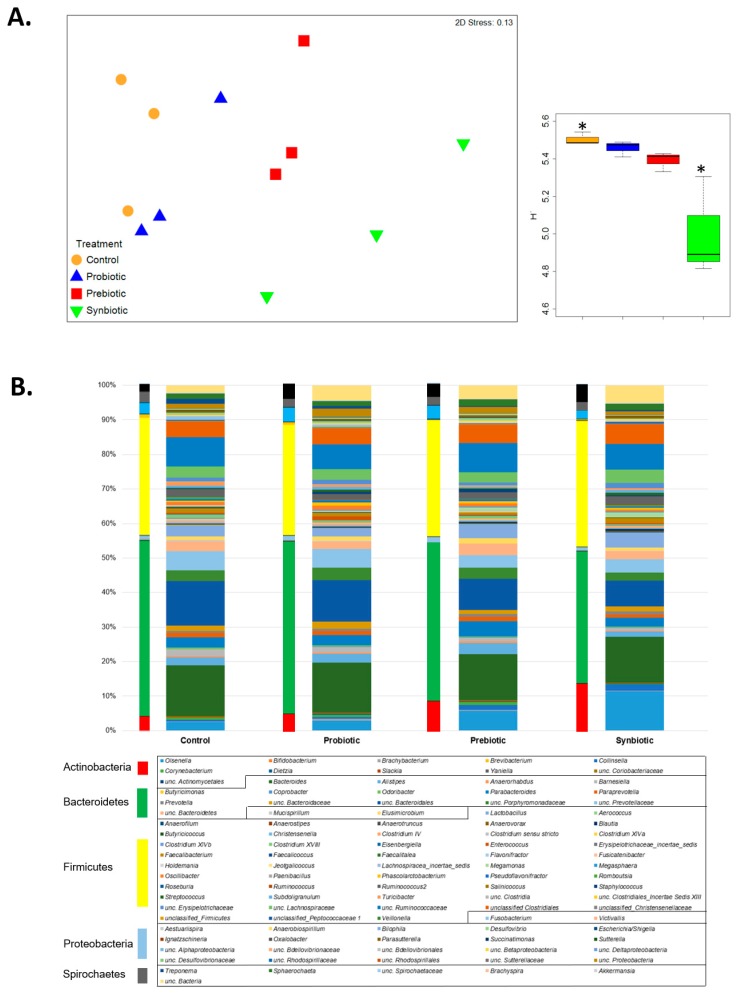
(**A**) Non-metric multidimensional scaling (nMDS) plot showing the distribution of the biological replicates on the diets. The diversity calculated with the Shannon index is plotted in the boxplot on the right bottom side. (**B**) Taxonomical information related to the 16S rRNA gene amplicon sequencing. The thinner bars indicate information at the phylum level and the wider bars indicate information at the genus level (average relative abundance >1%).

Exploring the global bacterial community structure through an nMDS plot, revealed the biological replicates grouped per diet. In this context, a clear separation was observed between the samples from the control and the probiotic, regarding those from the prebiotic and the synbiotic (Figure 1A). The average similarity within the replicates of the ceca samples was 67% in the control diet, 69% in prebiotic and probiotic, and 59% in synbiotic. The lowest Pielou’s evenness and Shannon diversity were found in the synbiotic diet (0.752 and 3.375, respectively) in comparison to the control (0.824 and 5.504, respectively; *p* < 0.030). No differences were observed between the remaining diets (Figure 1A), which was due to an increase of some specific microbial groups, at the expense of a variety of others. These results could imply less richness of the ceca microbiome, due to the synbiotic feeding, which was undesirable, due to the negative impact on poultry performance [10].

Bacteroidetes was the most abundant phylum identified in all diets, showing a similar abundance between the control diet and the probiotic (51% and 50% on average, respectively), while it presented relative lower values in prebiotic and synbiotic diets (46% and 38% on average, respectively; Figure 1B). Firmicutes was the following most abundant phylum, with similar values in all diets (34% on average; Figure 1B). In synbiotic, the abundance of Actinobacteria was more prominent (14% on average), in relation to the control diet (4% on average), prebiotic (9% on average), and probiotic (5% on average), while Proteobacteria was less abundant (2.5% on average), in comparison to the control diet (3.5% on average), prebiotic (4.0% on average), and probiotic (4.3% on average). At the genus level, Bacteroides was the most predominant across diets, with values between 13% and 15% (Figure 1B). In prebiotic and synbiotic diets, *Olsenella* was present in a higher abundance (6% and 11.5% on average, respectively), whereas, it was detected in a lower abundance in the control and the probiotic diets (2.5% and 3% on average, respectively). *Parabacteroides* was detected in the prebiotics, with 4.4% of abundance, in the control and the probiotic with 3%, and in the synbiotic diet with 2.6% of abundance. *Lactobacillus* was present in a higher abundance in the prebiotic and the synbiotic diets (4.1% and 4.4% on average, respectively), while it was lower in the control and the probiotic (3.0% and 2.5% on average, respectively).

PERMANOVA analysis indicated that feeding hens with prebiotics and synbiotics caused a different microbiota composition, compared to the control, whereas, feeding with a probiotic diet resulted in a similar structure (*p* < 0.001; Table 1). Prebiotic outcomes confirmed the prebiotic-modulatory effect on the ceca microbiome of poultry [5]. However, the probiotic results were unexpected because of the well-known repercussion of those in the ceca microbial communities [24]. A possible explanation for this finding was attributed to the late supplementation of the probiotic, which did not have the expected microbial modulation in the gut of the hens. Thus, it might be necessary to start with the dietary treatment, at the early stages of age, when the ceca microbiota is still in development [5]. Moreover, it could also be inferred that the type of probiotic evaluated, at the tested doses, was not the proper one to cause some change in the microbial composition.

**Figure 2:**
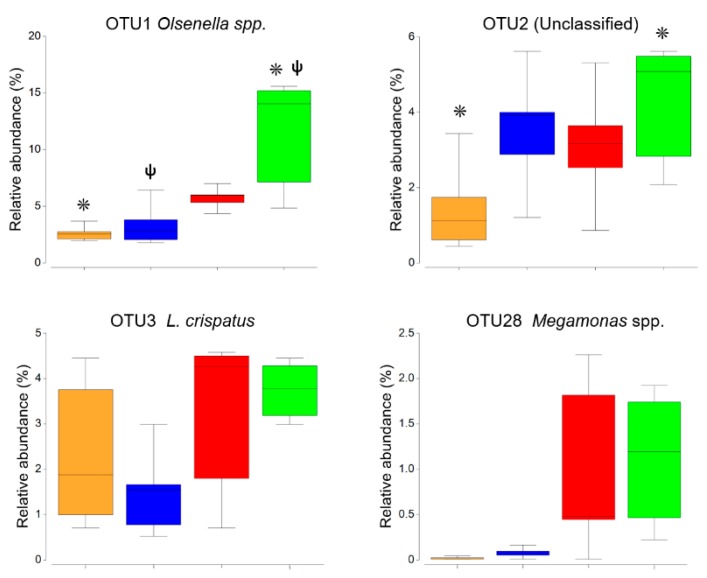
Box-plots with the relative abundance of the most abundant operational taxonomic units (OTUs). The color code indicates, yellow for control, blue for the probiotic, red for the prebiotic, and green for the synbiotic treatment. The symbols indicate statistical significance (*p* ≤ 0.05).

The percentage of dissimilarity of the ceca microbiota from hens fed with prebiotic and control was 46%, between prebiotic and probiotic it was 38%, between synbiotic and control it was 39%, and between synbiotic and probiotic it was 43%. These results were mainly due to the higher abundance of OTUs associated with the *Olsenella* spp. (OTU 1), and the *Lactobacillus crispatus* (OTU 3) in prebiotic and synbiotic diets (Figure 2). Both diets increased the abundance of OTU 1, which is an anaerobic bacterium that ferments carbohydrates to lactic acid. *Olsenella* spp., has been identified in the GIT of laying pullets being involved in lipid and cholesterol metabolism [25]. On the other hand, the observed increase of *L. crispatus* (OTU 3) could be considered desirable, because this phylotype has been classified as a beneficial probiotic, in poultry [26]. Due to its lactic acid production and competitive exclusion, supplementation of *L. crispatus* in broiler diets, reduces the colonization of *Campylobacter jejuni* and exerts an inhibitory effect against the presence of *Salmonella enterica* serovar Enteritidis [27]. With a lower relative abundance overall, a propionic acid bacterium identified as *Megamonas* spp. (OTU 28; [28]), increased in abundance, when feeding the diets mentioned above. This bacterium acts as a hydrogen sink in the ceca, increasing the short chain fatty acids (SCFAs) production, which brings benefits to the energy metabolism of the host [1].

### 3.2. Metagenomic Analysis

**Figure 3:**
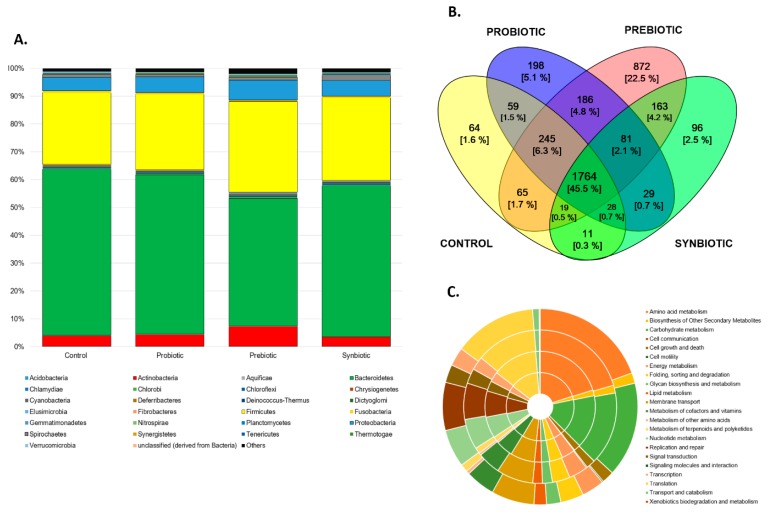
(**A**) Taxonomical assignation based on the KEGG protein database. The groups are shown at the phylum level. (**B**) Venn diagram depicting the percentage of genes assigned and shared between the four metagenomes (the color convention indicates, yellow for control, blue for the probiotic, red for the prebiotic, and green for the synbiotic. (**C**) KEGG orthologs obtained from the four metagenomes. The classification corresponds to the second level, and it was plotted as follows—the inner circle corresponds to the control, the second to the probiotic, the third to the prebiotic, and the outer circle corresponds to the synbiotic diet.

Taxonomic assignations of the metagenomic reads showed that the main phyla in all diets were Bacteroidetes (between 46% and 60%) and Firmicutes (26–33%), while less than 8% of the reads belonged to Actinobacteria and Proteobacteria (Figure 3A). This fact was in accordance with the study reported in laying hens from Bennett et al. [29], where Bacteroidetes was the dominant phylum, followed by Firmicutes. The reads were annotated using the KEGG database, and 4265 total genes were identified. They encoded microbial functions, and a core of 1764 (45.5%) was identified in the metagenomes (Figure 3B).

These findings indicated that diverse taxon’s in the ceca microbiota maintained a conserved core of genes, which was in agreement with Segerman [30]. However, microbial communities of the supplemented diets encoded for more unique functions, in comparison to the control. Paired analysis showed that all supplemented diets increased the microbial genes related to starch, sucrose, pyruvate, and glycerophospholipids metabolism, while the control diet mainly increased the microbial pathways for lysine degradation (Table 2), indicating a high activity of the central energy metabolism in the supplemented treatments. 

**Figure 4:**
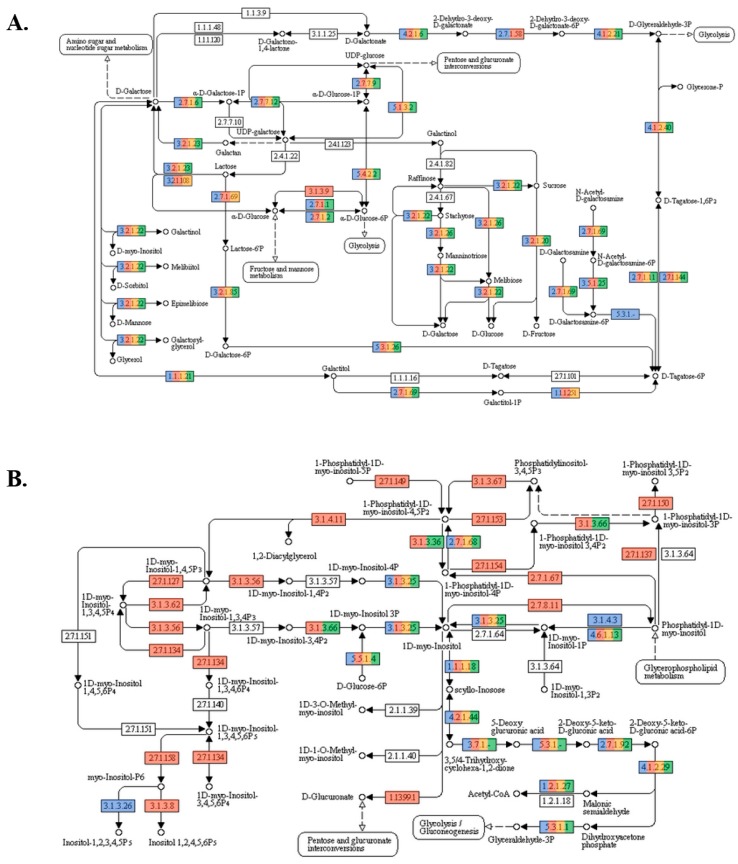
Metabolic pathways obtained from the KEGG mapper for: (**A**) Galactose metabolism and (**B**) Inositol phosphate metabolism. The color code indicates, respectively—yellow for control, red for the prebiotic, blue for the probiotic, and green for the synbiotic.

An increase in the expression of genes related to butanoate and propanoate metabolism (Table 2) was found in the prebiotic supplementation. These short chain fatty acids (SCFA’s) represent several beneficial effects to the host, related to mineral metabolism, the maintenance of sanitary status, and the energy metabolism. Their absorption by ceca mucosa provided up to 11% of the metabolizable energy, for mature birds [31,32], which could be used for productive purposes. In fact, laying hens fed with this prebiotic, showed a higher egg production, in comparison to the other diets [19]. Moreover, the galactose metabolism, which resulted in the hydrolysis of the lactose to the SCFAs, increased in the prebiotic diet (Figure 4A), probably exerted by the lactose from dry whey powder, as the only feed additive in the prebiotic diet.

Synbiotic supplementation augmented the pathways for starch and sucrose metabolism, in comparison to the others (Table 2). This treatment also increased the abundance of genes related to retinol and the glycolysis/gluconeogenesis metabolism, and the steroid hormone biosynthesis, in comparison to other diets. More pathways for amino sugar and nucleotide sugar metabolites were promoted by the symbiotic, compared to the probiotic and the prebiotic, as well as for the nicotinate (B3) and nicotinamide, with control.

Regarding the mineral metabolism, prebiotic increased the abundance of inositol phosphate (InsP) metabolism-related genes (Figure 4B), which was in agreement with previous reports, indicating that the degrading activities of InsP were mainly carried out in the ceca of laying hens [34]. Our results supported the role of the cecal microorganisms in the InsP degradation and phosphorous release, which was previously reported by Rodehutscord and Rosenfelder [35]. It was a positive finding for the laying hens because more phosphorus availability was desirable for the eggshell formation, hen skeletal integrity, and bone mineralization [36,37].

With regards to antibiotic resistance, it was interestingly observed that supplemented diets exhibited less of a presence of β-lactams resistance-related genes (Table 2). Specifically, our results showed less abundance of methicillin-resistant protein-related genes, in prebiotic and probiotic supplementation, and an absence of it in synbiotic supplementation, suggesting that the tested additives interfere with the presence of bacteria that harbor this resistance gene pools. However, the reasons why the additives might have inhibited the transfer of antibiotic resistance genes, are unclear. Nonetheless, it is a relevant finding because antibiotic resistance is a well-known threat to global animal and human health, compromising the therapeutic effectiveness of antibiotics in veterinary and human medicine [38]. Specifically, methicillin belongs to the penicillin antibiotic family, and, in an animal medicine context, it is widely used for treating the mainly staphylococcal infections in poultry [39].

Thus, prebiotic and synbiotic supplementation have a modulatory impact on the microbiota composition, while feeding laying hens with a probiotic or a basal diet, showed similar bacterial structures. All dietary supplementations induced modulations in the abundance or specific presence of microbial functional genes, although these did not imply a disturbance in their central biological roles.

## Figures and Tables

**Table 1:** Statistical differences between the diets and the main OTUs contributing to the cecal taxonomical differences in the four metagenomes. ^1^ Control—no additive supplementation; Probiotic—2 g/kg of P. acidilactici; Prebiotic—60 g/kg of dry whey powder, Synbiotic—2 g/kg of P. acidilactici and 60 g/kg of dry whey powder. Each line shows the results of the comparison between each diet (the Diet column) and each of the supplemented diets (the Groups column). *p* (perm) values ≤ 0.05 between comparisons were considered to be significantly different.

Statistical Differences between Diets Based on the PERMANOVA Results			
Diets ^1^	Groups	*t*-Value	*p* (perm)
Control	Probiotic	12.865	0.106
	Prebiotic	1.657	0.001
	Synbiotic	16.105	0.001
Probiotic	Prebiotic	16.325	0.001
	Synbiotic	14.991	0.001
Prebiotic	Synbiotic	1.225	0.190

**Table 2:** Genes differentially present based on the dietary treatment assigned. The difference was calculated with the log2fold change (LFC), after read normalization, using DESeq2 R package (Love et al., 2014) [33]. A cut-off criterion was established, which was *p* < 0.05 (Wald test). Positive or negative values indicate a higher or lower presence, respectively, of the assigned function, for the first mention treatment.

	Function	Control vs. Probiotic	Control vs. Prebiotic	Control vs. Synbiotic	Prebiotic vs. Probiotic	Prebiotic vs. Synbiotic	Probiotic vs. Synbiotic
Starch and sucrose metabolism	Beta-phosphoglucomutase [K01838]	1.64	1.72	2.33			
	Sucrose phosphorylase [K00690]			−1.82			
	Maltose phosphorylase [K00691]			−1.11			
	Maltose-6’-phosphate glucosidase [K01232]			−1.03			
	Beta-phosphoglucomutase [K01838]			−2.34			
	Cyclomaltodextrinase [K01208]			−1.23		−2.32	
	Glucan endo-1,3-beta-d-glucosidase [K01199]			−1.69		−3.05	−2.11
	Cellulose synthase (UDP-forming) [K00694]					−2.37	
Glycolisis/gluconeogenesis	Pyruvate decarboxylase [K01568]			−1.1			−2.12
	Acetyl-CoA synthetase (ADP-forming) [K01905]			−2.1		−1.47	−3.17
Piruvate metabolism	D-lactate dehydrogenase (cytochrome) [K00102]	3.48	4.13	3.69			
Citrate cycle (TCA cycle)	2-methylisocitrate dehydratase [K01682]	1.4	2.05	1.84			
Glycerophospholipid metabolism	Glycerol-3-phosphate dehydrogenase subunit B [K00112]	1.88	1.12	2.69			
Glycine, serine and threonine metabolism	Glycine amidinotransferase [K00613]	1.38	1.88	2.56			
	Cystathionine gamma-lyase [K01758]	1.63	1.63	2.56			
	Glycine N-methyltransferase [K00552]	Not in Ctrl	−1	Not in Syn	Not in Prob	Not in Syn	
	Creatinase [K08688]	−1	−1	Not in Syn			
	Diaminobutyric acid acetyltransferase[K06718]	−1	−1	Not in Syn			
	Cetaine-homocysteine S-methyltransferase [K00544]	Not in Prob	−1	Not in Syn			
	Choline dehydrogenase [K00108]	−2.11			1.23	3.81	
Arginine and proline metabolism	Creatinine amidohydrolase [K01470]	1.2	1.44	1.52			
	Ornithine decarboxilase [K01581]	1.8	1.22	1.89			
Cysteine metabolism	Aromatic-amino-acid transaminase [K00832]	1.21	1.37	2.18			
Cysteine and methionine metabolism	Homocysteine S-methyltransferase [K00547]			−1.76			
	Aromatic-amino-acid transaminase [K00832]			−2.18			
	(R)-2-hydroxyacid dehydrogenase [K05884]			−1.69		−1.05	
Tyrosine metabolism	2-hydroxyhepta-2,4-diene-1,7-dioate isomerase [K05921]	1.99	1.22	2.1			
Pantothenate and CoA biosynthesis	Type I pantothenate kinase [K00867]	1.02	1.2	1.68			
	Phosphopantothenoylcysteine decarboxylase [K01598]	1.79	1.8	1.1			
Folate biosynthesis	Para-aminobenzoate synthetase [K01247]	3.57	3.72	2.1			
Riboflavin metabolism	Low molecular weight phosphotyrosine protein Phosphatase [K14394]	1.31	1.22	3.1			
Lysine degradation	5-aminovalerate aminotransferase [K14268]	Not in Prob	Not in Preb	Not in Syn			
	Glutarate semialdehyde dehydrogenase [K14269]	Not in Prob	Not in Preb	Not in Syn			
β-lactam resistance	Methicillin resistance protein [K02547]	−2.82	−2.17	Not in Syn			
Butanoate metabolism	Glutaconate CoA-transferase, subunit A [K01039]		−1.4		Not in Prob	2.62	
	4-hydroxybutyrate dehydrogenase [K00043]		/		1.09	2.44	
	3-hydroxybutyrate dehydrogenase [K00019]		Not in Ctrl		Not in Prob	Not in Syn	
	Butanediol dehydrogenase/diacetyl reductase [K00004]		Not in Ctrl		Not in Prob	Not in Syn	
Propanoate metabolism	1-aminocyclopropane-1-carboxylate deaminase [K01505]		−1.22		/	1.8	
	2-methylcitrate dehydratase [K05608]		−1.57		1.61	Not in Syn	
	1-aminocyclopropane-1-carboxylate deaminase [K00923]		−1.22		/	1.7	
	2-methylcitrate synthase [K01659]		/		/	1.73	
	Methylisocitrate lyase [K03417]		/		/	2.44	
Fructose and mannose metabolism	mannitol 2-dehydrogenase [K00045]		/		/	1.19	
	PFK; 6-phosphofructo-2-kinase [K00900]		Not in Ctrl		1.01	1.7	
	Fructan beta-fructosidase [K03332]		−1.63		1.32	Not in Syn	
Inositol phosphate metabolism	5-dehydro-2-deoxygluconokinase [K03338]		−1.06		1.2	Not in Syn	
	iolB; 5-deoxy-glucuronate isomerase [K03337]		Not in Ctrl		1.76	1.7	
Galactose metabolism	Lactase-phlorizin hydrolase [K01229]		−3.22		2.23	Not in Syn	
	Galactitol-1-phosphate 5-dehydrogenase [K00094]		−1.37		2.38	Not in Syn	
	Tagatose 6-phosphate kinase [K00917]		/		/	1.17	
	2-dehydro-3-deoxyphosphogalactonate aldolase [K01631]		/		2.97	1.12	
	Galactonate dehydratase [K01684]		/		/	1.35	
	Maltase-glucoamylase [K12047]		Not in Ctrl		Not in Prob	Not in Syn	
	*N*-acetylgalactosamine-6-phosphate deacetylase [K02079]			Not in Ctrl		Not in Preb	Not in Prob
Phenylalanine, tyrosine and tryptophan biosynthesis	Anthranilate synthase/phosphoribosyltransferase [K13497]		−4.96		1.8	/	
	Indole-3-glycerol phosphate synthase/Phosphoribosylanthranilate isomerase [K13498]		−1.42		1.26	1.26	
	Shikimate kinase/3-dehydroquinate synthase [K13829]		−1.09		1.78	2.99	
	Chorismate mutase/prephenate dehydratase [K14170]		/		/	1.27	
	Cyclohexadienyl dehydratase [K01713]			Not in Ctrl		Not in Preb	Not in Prob
Fatty acid metabolism	Long-chain-fatty-acid-[acyl-carrier-protein] Ligase [K05939]		−2.17		1.55	/	
	Cytochrome P450, family 4, subfamily A [K07425]		Not in Ctrl		Not in Prob	Not in Syn	
	Carnitine O-palmitoyltransferase 2 [K08766]		Not in Ctrl		Not in Prob	Not in Syn	
Fatty acid biosynthesis	Fatty acid synthase, bacteria type [K11533]		/		1.22	4.56	
	Oleoyl-[acyl-carrier-protein] hydrolase [K01071]			−1.7		/	−1.24
Thiamine metabolism	Hydroxyethylthiazole kinase [K14154]		/		2.23	1.11	
	Thiamine-phosphate diphosphorylase [K14153]		−1.22		/	Not in Syn	
Retinol metabolism	Retinol dehydrogenase 16 [K11154]		Not in Ctrl		Not in Prob	Not in Syn	
	Diacylglycerol O-acyltransferase 2-like protein 4 [K11156]		Not in Ctrl		Not in Prob	Not in Syn	
	All-trans-retinol 13,14-reductase [K09516]			−1.1		−1.76	−1.83
Ascorbate and aldarate metabolism	D-threo-aldose 1-dehydrogenase [K00064]	−1.23			/		2.13
	l-ribulose-5-phosphate 3-epimerase [K03079]	−1.1			−3.05		1.58
Glyoxylate and dicarboxylate metabolism	Formate dehydrogenase [K00122]	Not in Ctrl			−2.94		/
	Oxalyl-CoA decarboxylase [K01577]	/			−2.4		1.07
	N,N-dimethylformamidase [K03418]	Not in Ctrl			Not in Preb		Not in Syn
	Formate dehydrogenase-N, gamma subunit [K04509]	Not in Ctrl			Not in Preb		Not in Syn
	Glycolate oxidase FAD binding subunit	Not in Ctrl			Not in Preb		Not in Syn
	Oxalate decarboxylase [K01569]	Not in Ctrl			Not in Preb		Not in Syn
	Malate dehydrogenase [K00025]	Not in Ctrl			−0.15		Not in Syn
Alanine, aspartate and glutamate metabolism	Aspartate 4-decarboxylase [K09758]	/			−1.17		2.61
	1-pyrroline-5-carboxylate dehydrogenase [K00294]	/			/		1.01
	Carbamoyl-phosphate synthase [K01954]	/			−1.27		1.97
	Delta 1-pyrroline-5-carboxylate dehydrogenase [K13821]	−1.59			/		2.3
Phenylalanine metabolism	Phenylacetaldehyde dehydrogenase [K00146]	−2.47			−2.94		/
	2-keto-4-pentenoate hydratase [K02554]	−1.57			−1.94		/
	Cinnamic acid dioxygenase subunit alpha [K05708]	−1.31			−2.67		/
Nicotinate and nicotinamide metabolism	UDP-sugar diphosphatase [K11751]	−1.49			/		/
	5′-nucleotidase [K01081]	/			−1.05		/
	NAD(P) transhydrogenase [K00322]	/			−1.81		/
	Nicotinamide-nucleotide adenylyltransferase [K00952]	/			−3.16		/
	Purine nucleosidase [K01239]			−1.08		/	/
	NAD(P) transhydrogenase subunit alpha [K00324]			−1.1		/	/
Amino sugar and nucleotide sugar metabolism	Chitin deacetylase [K01452]			/		−2.47	−2.12
Valine, leucine and isoleucine biosynthesis	Valine—pyruvate aminotransferase [K00835]			−3.69		−4.05	/
Steroid hormone biosynthesis	Steroid delta-isomerase [K01822]			/		−1.68	−1.17
	Steryl-sulfatase [K01131]			−1.1		−2.45	−2.12
